# Predictive Factors for Spontaneous Resolution in Primary Obstructive Megaureter: The Impact of Hydronephrosis Severity on Clinical Outcomes

**DOI:** 10.3390/jcm14072463

**Published:** 2025-04-04

**Authors:** George Vlad Isac, Nicolae Sebastian Ionescu

**Affiliations:** 1 Department of Pediatric Surgery and Orthopedics, “Carol Davila” University of Medicine and Pharmacy, 050474 Bucharest, Romania; sebastian.ionescu@umfcd.ro; 2 Department of Pediatric Surgery, “M.S. Curie” Clinical Emergency Hospital for Children, 077120 Bucharest, Romania; 3 The Academy of Romanian Scientists, 050044 Bucharest, Romania; 4 Romanian Academy of Medical Sciences, 030167 Bucharest, Romania

**Keywords:** primary obstructive megaureter, conservative treatment, spontaneous resolution, predictive factors, hydronephrosis severity, pediatric urology

## Abstract

**Background/Objectives:** Primary obstructive megaureter (POM) is a rare congenital urological condition usually diagnosed prenatally or in early childhood. Conservative management is increasingly preferred due to a high potential for spontaneous resolution. However, reliable predictors of spontaneous resolution remain controversial, complicating clinical decision-making. This study aimed to identify the demographic, clinical, and imaging parameters predictive of spontaneous resolution in patients with primary obstructive megaureter. **Methods:** We retrospectively analyzed 21 pediatric patients diagnosed with primary obstructive megaureter, who were treated conservatively at the Maria Sklodowska Curie Emergency Clinical Hospital for Children from January 2015 to December 2024. Clinical parameters, imaging findings, and renal function were evaluated. Statistical analyses included univariate comparisons and multivariate logistic regression modeling. **Results:** Spontaneous resolution occurred in 12 (57%) patients, at a median age of 45.75 months. The only statistically significant predictor identified was the initial hydronephrosis grade (*p* = 0.046). Patients with mild-to-moderate dilation (Grades I–II) had a significantly higher resolution rate (11 of 15 cases) compared with those with severe dilation (1 of 6 cases). Ureteral diameter showed a trend toward predicting outcomes, with unresolved cases having larger median diameters (15 mm vs. 10.5 mm, *p* ≈ 0.08). Age at diagnosis, sex, bilateral involvement, and history of urinary infections did not significantly influence resolution rates. **Conclusions:** The initial severity of hydronephrosis significantly predicts spontaneous resolution in primary obstructive megaureter. Conservative management is particularly justified in mild-to-moderate cases, whereas patients with severe dilation may require early intervention due to lower resolution likelihood.

## 1. Introduction

First described by Caulk in 1923, the term megaloureter originally referred to a dilated ureter without hydronephrosis. Over time, the terminology evolved to megaureter, now classified into four primary types—obstructive, refluxing, combined obstructive/refluxing, and non-obstructive/non-refluxing. This classification, introduced in the 1970s, remains the standard framework for distinguishing the etiology and clinical implications of megaureter [1,2].

A normal ureter rarely exceeds 5 mm in diameter, whereas ≥7 mm is considered diagnostic for megaureter. This condition may be primary (idiopathic) or secondary to underlying conditions such as posterior urethral valves, vesicoureteral reflux (VUR), neurogenic bladder, outlet obstruction, or infection. Identifying secondary causes is crucial, as management should address the underlying pathology rather than its consequences [1,2].

Primary obstructive megaureter is defined as a congenital dilation of the ureter exceeding 7 mm due to functional or anatomical obstruction at the ureterovesical junction. This obstruction prevents normal urinary flow, leading to urine stasis, ureteral dilation, and potential renal impairment [2].

One of the underlying mechanisms in POM is functional obstruction near the vesicoureteral junction (VUJ), often caused by an aperistaltic ureteral segment usually measuring 0.5–4 cm in length. This segment lacks intrinsic peristaltic activity, impairing urine propulsion into the bladder, which results in progressive ureteral dilation. In addition, transient functional or anatomical factors may contribute to early postnatal dilation. These include persistent fetal ureteric folds, which can temporarily obstruct urine flow and delayed peristaltic maturation, impairing urine propulsion. These transient obstructions often resolve spontaneously as the child grows, leading to improvement or the complete resolution of megaureter in some cases [3].

Histologically, primary obstructive megaureter may exhibit a focal deficiency of muscle fibers in the ureterovesical segment, with proximal muscular hypertrophy, an abnormal predominance of circular fibers within the intramural ureter, marked collagen infiltration, and varying degrees of distal muscular dysplasia [4,5].

Electronic microscopy has revealed increased collagen deposits between the lamina propria and the smooth muscle fibers, as well as localized muscular alterations strictly confined to the ureterovesical junction [5,6].

Some forms of obstructive megaureter may show spontaneous improvement during the first years of life. This favorable evolution appears to correlate with the segmental maturation of the distal ureter and a decrease in the expression of transforming growth factor beta (TGF-β), which is implicated in delayed smooth muscle differentiation. TGF-β has been detected in children under two years of age with obstructive uropathy, but not in those who underwent surgery for non-obstructive conditions [7,8].

The clinical presentation of POM is highly variable, depending on patient age at diagnosis, the severity of the obstruction, and the presence of associated anomalies. In neonates and infants, POM is often detected prenatally during routine ultrasound screenings. Postnatal ultrasound may reveal ureteral dilation and hydronephrosis, prompting further evaluation [9]. Postnatally, affected infants may present with symptoms such as urinary tract infections (UTIs), which are a common complication due to the stasis of urine in the dilated ureter. Febrile UTIs are particularly concerning and have been identified as a significant risk factor in children with ureteral dilation greater than 11 mm. As children grow older, the clinical presentation may vary. Some children may remain asymptomatic, while others may experience recurrent UTIs, abdominal pain, or hematuria. The abdominal pain can be a direct consequence of the obstructive nature of the megaureter, leading to discomfort and potential complications such as renal stone formation. Microscopic hematuria is frequently observed in the absence of infection and is presumably caused by the disruption of mucosal vessels of the ureter secondary to ureteric distension. Hematuria may also indicate calculus formation due to urinary stasis. The degree of ureteral obstruction plays a crucial role in the symptomatology. Severe obstructions are more likely to cause significant symptoms and complications, whereas mild obstructions may remain asymptomatic and be discovered incidentally during imaging studies for other reasons [3,10,11].

Given this variability, individualized assessment and monitoring are essential, considering factors such as the degree of ureteral dilation, the presence of associated anomalies, and renal function impairment. Close follow-up is critical to prevent complications and ensure appropriate intervention when necessary [12].

Historically, surgical intervention was the standard of care for POM. However, recent studies suggest that a significant proportion of cases resolve spontaneously, leading to an increased preference for conservative management [13,14].

Clinicians guide the management of POM based on the severity of symptoms and the extent of renal impairment. For asymptomatic patients with mild obstruction, they may choose conservative management—including antibiotic prophylaxis and regular monitoring—but often recommend surgical intervention for patients with significant obstruction or recurrent infections.

Spontaneous resolution rates vary widely, ranging from 34% to 88%, depending on baseline ureteral diameter (a dilation < 11 mm is predictive of resolution, while ≥14 mm suggests a higher likelihood of surgery) and the severity of hydronephrosis (higher SFU grades are associated with lower resolution rates) [15,16].

Regular follow-up is necessary to assess the patient’s condition during the observation period. This includes periodic imaging studies such as ultrasound and scintigraphy scans to evaluate the degree of hydronephrosis and ureteral dilation. The primary goal of observation is to preserve upper urinary tract function while avoiding unnecessary surgical interventions. This approach is particularly beneficial in infants and young children, where the risks associated with surgery may outweigh the potential benefits. However, it is essential to balance the risks of prolonged obstruction, which can lead to renal damage, against the potential for spontaneous resolution [17].

Despite these findings, clinical decision-making remains challenging due to high variability in presentation and progression. Accurately predicting which patients will resolve spontaneously versus which will require surgery is critical to avoid unnecessary long-term monitoring, reduce the risk of recurrent UTIs and renal damage, and prevent delayed surgical intervention in high-risk cases.

Previous studies have explored several potential predictors, including the severity of hydronephrosis, ureteral diameter, renal parenchymal thickness, and the presence of bilateral disease clinical symptoms, but existing predictive models are inconsistent [13,14].

By analyzing data from pediatric patients managed conservatively at the Maria Sklodowska Curie Emergency Clinical Hospital for Children, Bucharest, Romania, we aimed to improve risk stratification criteria for conservative management, enhance clinical decision-making regarding the need for surgery, and optimize follow-up strategies to reduce unnecessary interventions.

This study sought to provide clinically relevant insights that will guide patient management, ensuring better outcomes and cost-effective care.

## 2. Materials and Methods

### 2.1. Study Design

This is a single-center, observational retrospective cohort study conducted over 10 years to evaluate predictors of spontaneous resolution in primary obstructive megaureter. It was carried out at the Department of Pediatric Surgery at the Maria Sklodowska Curie Emergency Clinical Hospital for Children, Bucharest, Romania. The study included patients diagnosed and managed conservatively and monitored over time. Data were collected from January 2015 to December 2024 from hospital medical records and follow-up visits.

### 2.2. Patient Selection and Exclusion Criteria

Patients were identified based on their medical records and imaging findings, with inclusion criteria as follows: age between 0 and 18 years; diagnosis of obstructive megaureter (distal ureter > 7 mm diameter) confirmed through ultrasonography and Uro-CT/Uro-MRI or nuclear renal scintigraphy; the absence of vesicoureteral reflux on voiding cystourethrography (VCUG)/contrast-enhanced voiding urosonography; initial management with conservative follow-up, without early surgical intervention; available follow-up data for at least 6 months.

The exclusion criteria were as follows: patients with secondary causes of megaureter (spina bifida/neurogenic bladder, posterior urethral valves, vesicoureteral reflux, and ureterocele); insufficient clinical or imaging data in hospital records (2 cases); patients lost to follow-up (3 cases).

After applying these criteria, 21 patients were included in the final analysis.

### 2.3. Diagnostic and Follow-Up Methods

All patients underwent ultrasound and voiding cystourethrography as initial diagnostic tools. In cases with inconclusive ultrasound findings, we complemented the evaluation with CT urography or MR urography. We also performed DTPA (diethylenetriamine pentaacetate) renal scintigraphy whenever possible, ensuring comprehensive diagnostic accuracy. Conservative management included regular monitoring through clinical follow-up, imaging, and, when necessary (recurrent UTIs history, ureteral diameter over 10 mm), antibiotic prophylaxis.

Ultrasound measurements of the anteroposterior renal pelvis diameter, renal parenchyma, and ureteral diameter were obtained using a standardized protocol with a full bladder.

The diuretic scintigraphy was typically performed after 3 months of life, under standardized hydration and bladder emptying conditions. According to the guidelines of the European Association of Urology (EAU), oral hydration was administered before the scan, followed by an intravenous infusion of saline (15 mL/kg over 30 min) administered 15 min prior to radionuclide injection, and maintained throughout the procedure with a continuous infusion (4 mL/kg/h).

Furosemide administration (1 mg/kg for infants under 1 year of age, 0.5 mg/kg for children aged 1–16 years, with a maximum dose of 40 mg) is recommended 20 min after isotope injection to help differentiate obstructive from non-obstructive patterns. Key parameters assessed include differential renal uptake, time to peak activity, and half-time (T½) after diuretic administration [18].

Follow-up assessments were performed every 3–6 months during the first year and annually thereafter, allowing us to monitor patient evolution over the 10-year study period and confirm the absence of external interventions that might have influenced the outcome.

### 2.4. Data Collection and Variables

Demographic, clinical, imaging, and functional data were collected from patient records. The variables analyzed included the following: age at diagnosis (months), sex, and residence (urban/rural); antenatal vs. postnatal diagnosis, a history of urinary tract infections, symptoms at presentation, and antibiotic prophylaxis; laboratory tests (seric urea and creatinine, urinalysis); the degree of hydronephrosis (Society for Fetal Urology grading system), maximum anteroposterior renal pelvis diameter, maximum distal ureteral diameter, and renal parenchymal thickness; differential renal function (DRF, measured via DTPA scintigraphy) and the presence of an obstruction on diuretic renography (T½ > 20 min); time to spontaneous resolution (months), last documented follow-up, and the need for surgical intervention.

Data were collected and organized in a structured database using Microsoft Office 2021. ChatGPT-4 was used to assist in translating sections of the manuscript from Romanian to English. The translation was subsequently reviewed and refined using Grammarly to ensure clarity, grammar accuracy, and adherence to academic writing standards.

### 2.5. Outcome Definition

The primary outcome was spontaneous resolution of obstructive megaureter, defined as complete normalization or significant reduction (distal ureteral diameter < 7 mm) in ureteral dilation on two serial ultrasonography sessions (3–6-month intervals), and the normalization of the calyceal pattern, no evidence of progressive obstruction or impaired renal function on follow-up imaging, and no need for surgical intervention throughout the follow-up period. Clinically, we considered the absence of urinary symptoms (particularly febrile UTIs and abdominal pain), while laboratory findings included normal serum creatinine levels and negative urine cultures.

Patients who did not meet these criteria were classified as having persistent/stationary disease or progressive obstruction, requiring correction.

### 2.6. Statistical Analysis

Descriptive statistics were used to summarize patient characteristics. Continuous variables were expressed as median (interquartile range, IQR) or mean ± standard deviation (SD), depending on the data distribution. Categorical variables were reported as frequencies and percentages.

Comparative analysis between patients with and without spontaneous resolution was performed using the Mann–Whitney U test for non-normally distributed continuous variables, the independent *t*-test for normally distributed continuous variables, and the Chi-square test or Fisher’s exact test for categorical variables.

To identify independent predictors of spontaneous resolution, univariate and multivariate logistic regression was performed, calculating odds ratios (ORs) with 95% confidence intervals (CIs).

Variables for the multivariate logistic regression model were selected based on a combination of univariate analysis results (*p* < 0.2) and clinical relevance.

Statistical significance was set at *p* < 0.05. All analyses were performed using SPSS 20.0.

### 2.7. Ethical Considerations

This study was conducted in accordance with the Declaration of Helsinki and was approved by the Ethics Committee of the Maria Sklodowska Curie Emergency Clinical Hospital for Children (3216/23 January 2025). Informed consent was not required due to the retrospective nature of the study. However, upon admission to our institution, patients sign a consent form agreeing to participate in scientific activities, allowing the results of potential future studies to be published in the specialized literature. Patient confidentiality was strictly maintained, and data were anonymized before analysis.

## 3. Results

### 3.1. Descriptive Analysis

#### 3.1.1. Patient Characteristics

Our study included 21 pediatric patients diagnosed with primary obstructive megaureter, encompassing 28 affected renal units. The median age at diagnosis was 1 month (IQR: 0–45; range: 0–83 months). The median age at first hospital admission was 11 months (IQR: 2.2–51.3; range: 0.3–16.9). The cohort was predominantly male (*n* = 16, 76%), with five female patients (24%). Prenatal diagnosis was established in 9 patients (43%), while the remaining 12 (57%) were diagnosed postnatally based on clinical symptoms (58%) or incidental imaging findings (42%). The median weight at admission was 11.3 kg (range: 3.6–27 kg) (Table 1).

Clinically, eight patients (38%) experienced at least one urinary tract infection during follow-up, while five (24%) reported symptoms such as lumbar pain or hematuria; the remaining patients were asymptomatic. A total of 11 patients (52%) received antibiotic prophylaxis during monitoring. The average number of hospitalizations was 5.3 per patient.

#### 3.1.2. Imaging Findings

The condition was slightly more prevalent on the left side (*n* = 15, 53.6% of the affected renal units), although the distribution was relatively balanced. Bilateral involvement was observed in seven patients (33%).

Based on the Society for Fetal Urology (SFU) grading system, the severity of hydronephrosis was classified as follows:

- Grades I–II (mild-to-moderate hydronephrosis)—15 patients (71%);
- Grades III–IV (severe hydronephrosis)—6 patients (29%).

The median distal ureteral diameter was 11 mm for the left side (IQR: 8–13; range: 7–17 mm) and 10 mm for the right side (IQR: 9–15; range: 7–21 mm), while the median renal pelvis anteroposterior (AP) diameter was 11 mm on the left (IQR:8–14; range: 4–27) and 12 mm on the right (IQR: 9–16; range: 3–28 mm) (Table 1).

#### 3.1.3. Renal Functional Assessment

Renal function was evaluated using nuclear scintigraphy (DTPA scans) in 17 cases and the median glomerular filtration rate (GFR) was as follows:

- Left HUN—63 mL/min/1.73 m^2^ (IQR: 49–76; range: 36–108 mL/min/1.73 m^2^);
- Right HUN—55 mL/min/1.73 m^2^ (IQR: 47–78; range: 9–115 mL/min/1.73 m^2^) (Table 1).

Five patients (23.8%) demonstrated a prolonged T½ (>20 min), indicating significant obstruction. Only one case showed a DRF below 40%.

#### 3.1.4. Clinical Outcomes

The median follow-up duration was 60 months (IQR: 24–84; range: 3–114 months). Spontaneous resolution was observed in 12 patients (57%), as confirmed through serial imaging, which demonstrated reduced ureteral dilation below 7 mm and stable renal function. The median age at resolution was 32 months (IQR: 16–75; range: 12–107 months).

Persistent or worsening obstruction was noted in nine patients (43%), with a median ureteral diameter of 15 mm. Of these, five (24%) remained under active surveillance, as their condition remained stable without clinical or functional deterioration. Surgical intervention was required in four patients (19%) due to progressive ureteral dilation or worsening renal function (Table 1).

### 3.2. Interpretation of Statistical Analysis 

The analysis identified the initial grade of hydronephrosis as the only statistically significant factor distinguishing patients who achieved spontaneous resolution from those who did not. Specifically, patients with high-grade hydronephrosis (Grades III–IV) were significantly less likely to experience spontaneous resolution than those with low-grade dilation (Grades I–II). Only 1 out of 6 patients (16.7%) with high-grade dilation achieved resolution, compared with 11 out of 15 patients (73.3%) with low-grade megaureter (*p* = 0.046).

For other variables, no statistically significant differences were observed, although some trends emerged. The maximum ureteral diameter was, on average, larger in patients without spontaneous resolution (median: 15 mm) than in those who achieved resolution (median: 10.5 mm), suggesting a potential association between greater ureteral dilation and the need for intervention; however, this difference did not reach statistical significance (*p* ≈ 0.08). Similarly, the proportion of patients requiring antibiotic prophylaxis—used as a marker of infection risk—was higher in the unresolved group (78% vs. 33%, *p* = 0.06), indicating that patients with a less favorable clinical course may have had a higher infection risk.

Renal parenchymal thickness did not significantly differ between the resolved (median: 11 mm) and unresolved (median: 10.5 mm) groups (*p* = 0.91), indicating that this parameter did not influence spontaneous resolution.

Similarly, baseline renal function, assessed by creatinine levels, showed no significant difference between the two groups (median: 0.30 mg/dL in the resolved group vs. 0.31 mg/dL in the unresolved group).

Age at diagnosis did not impact resolution rates, suggesting that both antenatally diagnosed neonates and older children had similar outcomes in this cohort.

Additionally, sex, bilateral involvement, and UTI history during follow-up were not significantly associated with spontaneous resolution (*p* > 0.4 in all cases; *p* = 0.68 for UTI history).

To identify independent predictors of spontaneous resolution, a multivariate logistic regression analysis was performed. Due to the limited sample size and number of resolution events, only a select number of variables were included in the model, chosen based on their differences in the univariate analysis. The model assessed dilation grade (III–IV vs. I–II), ureteral diameter, symptom presence, and UTI history.

Among these predictors, the initial hydronephrosis grade remained the only significant independent factor associated with spontaneous resolution after adjusting for other variables. Specifically, patients with Grades III–IV had a significantly lower probability of spontaneous resolution than those with Grades I–II (OR: 0.07; 95% CI: 0.006–0.83; *p* ≈ 0.035), indicating a ~93% reduction in the odds of resolution for patients with severe dilation. Conversely, patients with Grade I–II megaureter were significantly more likely to achieve a favorable outcome under conservative management, independent of other factors.

Regarding ureteral diameter, the model suggested a trend toward an inverse association with spontaneous resolution (OR ≈ 0.82 per additional 1 mm increase in diameter), implying that a more dilated ureter may slightly reduce the likelihood of resolution. However, this association did not reach statistical significance in the multivariate regression analysis (*p* ≈ 0.2). The presence of clinical symptoms and UTI history did not independently contribute to the model after adjusting for anatomical severity.

Overall, the multivariate logistic regression analysis confirmed that the initial severity of obstruction, based on hydronephrosis grade, is an independent predictor of spontaneous resolution under conservative management. Other variables either did not demonstrate a statistically significant effect or were likely collinear with anatomical severity. For instance, patients with more severe dilation were also more likely to be symptomatic and to receive antibiotic prophylaxis, suggesting an overlap in the significance of these factors.

## 4. Discussion

A comparison with the literature highlights key predictive factors influencing the spontaneous resolution of primary obstructive megaureter. Various studies have explored the roles of patient age, the degree of obstruction, ureteral dilation, renal function, and associated anomalies in determining outcomes. Our findings align with these reports, particularly regarding the impact of hydronephrosis grade as a major determinant of spontaneous resolution.

Patient age has been previously identified as a significant predictor of POM resolution, with cumulative resolution rates increasing from 66% at 2 years to 97% after 6 years [19]. However, in our cohort, age at diagnosis was not significantly associated with spontaneous resolution. This suggests that while younger patients may generally have a higher likelihood of resolution, age alone does not independently predict outcomes when adjusted for hydronephrosis severity. Our findings contrast with some previous studies emphasizing the importance of patient age, but they align with others indicating that anatomical severity may play a more critical role in determining resolution likelihood [9].

The severity of obstruction emerged as the primary predictive factor for spontaneous resolution in our study, with patients presenting with high-grade hydronephrosis (Grades III–IV) being significantly less likely to resolve spontaneously (*p* = 0.046). This is consistent with Braga et al., who reported that 57% of patients with low-grade hydronephrosis achieved resolution, compared with only 29% of those with high-grade hydronephrosis [20]. Our logistic regression model confirmed this trend, demonstrating that the severity of hydronephrosis at presentation is a critical determinant of outcome under conservative management.

Our study did not identify renal parenchymal thickness as a significant predictor of spontaneous resolution, as median values were comparable between resolved and unresolved cases (*p* = 0.91). This finding contrasts with research suggesting that advanced hydronephrosis can lead to medullary pyramid compression and renal parenchymal atrophy, potentially affecting renal function and resolution likelihood [21]. Given the variability in reported findings, further research is needed to determine whether renal parenchymal changes influence long-term outcomes.

The distal ureteric diameter has been a point of contention, with varying opinions on its predictive value [22].

Although the ureteral diameter exhibited a trend toward an inverse association with spontaneous resolution (OR ≈ 0.82 per additional 1 mm increase in diameter), it did not reach statistical significance in our multivariate model (*p* ≈ 0.2). Previous studies have debated the role of ureteral diameter in predicting resolution. While some studies have found a weak correlation between larger ureteral diameter and prolonged resolution time, our results suggest that while ureteral diameter may be a contributing factor, its predictive value is not as strong as hydronephrosis grade.

Associated anomalies also influence the prognosis of POM. Concurrent conditions can complicate the clinical picture and affect the resolution rates. For instance, Drlík et al. found that patients without additional anomalies had better outcomes than those with associated conditions [23]. This highlights the need for a comprehensive evaluation of each patient to identify any coexisting anomalies that may impact the treatment strategy. Our study did not specifically assess congenital anomalies as independent variables in the regression model, but their potential influence should be considered in future research.

Additionally, histopathological studies suggest that excessive collagen deposition and segmental muscle dysfunction may contribute to functional obstruction in primary megaureter [24]. These structural abnormalities may explain why some severe cases do not resolve spontaneously, reinforcing the need for individualized patient management strategies.

The management practices and clinical guidelines for POM have evolved based on these predictive factors. Farrugia et al. discussed the development of consensus guidelines for managing primary megaureter, emphasizing the role of evidence-based practices in improving patient outcomes. These guidelines consider patient age, the degree of obstruction, and the presence of associated anomalies to tailor the management approach for each case [25].

The effectiveness of conservative therapy for megaureter has been a topic of ongoing discussion. Studies have indicated that organic obstruction of the terminal part of the ureter is found in a minority of cases, with the majority of urodynamic disturbances being functional [26]. This distinction is significant because functional obstructions may resolve spontaneously, after the maturation of the adynamic terminal ureter segment, whereas organic obstruction often requires surgical intervention.

Several limitations should be considered when interpreting our findings. The primary limitation is the relatively small sample size (*n* = 21), which reduces the statistical power to detect significant associations. This is particularly relevant for subgroup analyses and logistic regression models, where collinearity between variables (e.g., hydronephrosis severity and ureteral dilation) may confound results. Larger, multicenter studies are necessary to validate our findings and improve generalizability.

The rarity of primary obstructive megaureter as a clinical condition limited the number of cases we included in this study. Moreover, the study was conducted within a single tertiary referral center, a national center for complex pediatric urology cases. While this may limit the sample size, it also ensures high consistency in patient selection, clinical approach, diagnostic evaluation, imaging protocols, and follow-up strategies. By focusing the study within one institution, we reduced the heterogeneity often seen in multicenter research, where differences in diagnostic criteria, imaging techniques, treatment decisions, and monitoring approaches can compromise data comparability. These differences are particularly relevant without a universally accepted standardized protocol for conservatively managing primary obstructive megaureter.

Through this study, we aim to lay the groundwork for developing a standardized protocol to promote more consistent clinical practice and support future multicenter collaborations guided by unified criteria.

Additionally, the retrospective study design introduces inherent risks of selection bias and incomplete data retrieval. Variability in imaging modalities, follow-up intervals, and clinical decision-making among treating physicians may have influenced outcome assessments.

Furthermore, the reliance on radiological findings as a primary diagnostic tool presents its own set of challenges. Di Renzo et al. note that in young children, defining obstruction is particularly demanding, and some indices such as half-times, while sensitive, are not specific [9]. This can result in either the overestimation or underestimation of obstruction severity, impacting clinical decisions.

Differences in imaging techniques, such as magnetic resonance urography (MRU) versus scintigraphy, may also contribute to inconsistencies in hydronephrosis grading and resolution rates [27].

Long-term renal function outcomes were not systematically evaluated in this study. Previous research has suggested that even in cases of initial spontaneous resolution, functional deterioration can occur over time, emphasizing the need for extended follow-up [25]. Future studies should incorporate longitudinal renal function assessments to determine the true impact of spontaneous resolution versus persistent obstruction on renal health.

Another important consideration is the potential underestimation of surgical indications based on initial DRF measurements. While some reports suggest that low DRF should not automatically indicate early surgery, others emphasize the importance of early intervention in cases with declining function [23,28]. Integrating advanced imaging and functional markers into clinical decision algorithms may help refine surgical criteria.

Renal scarring is also a significant morphological predictor. The early detection of renal parenchyma sclerosis can lead to a more rational approach to renoprotective therapy, potentially slowing down or preventing the further progression of renal scarring [26]. This highlights the importance of early and accurate morphological assessment in managing POM. Furthermore, the anatomical stability of the affected renal units is a key factor. Gimpel et al. observed that kidneys with POM exhibited entirely normal longitudinal growth when compared with healthy contralateral renal units. This indicates that anatomical stability can be a positive predictor for spontaneous resolution, as long-term outcomes were excellent in these cases [22].

Although renal function (GFR) was assessed initially, we did not systematically investigate long-term renal function or potential renal scarring outcomes. This limits our understanding of how spontaneous resolution versus persistent obstruction impacts renal health over the long term.

## 5. Conclusions

Spontaneous resolution occurred in 57% of cases, at a median age of 45.75 months.

Hydronephrosis severity was the strongest independent predictor of spontaneous resolution for primary obstructive megaureter in our study. Under conservative management, patients with low-grade hydronephrosis (Grades I–II) had significantly higher resolution rates. By contrast, those with high-grade dilation (Grades III–IV) exhibited a 93% reduction in resolution odds and were more likely to require surgical intervention.

Ureteral diameter, renal function, and infection history did not independently predict spontaneous resolution. Although a trend was observed suggesting that more significant ureteral dilation may reduce the likelihood of resolution, statistical significance was not reached. Similarly, renal parenchymal thickness, baseline creatinine levels, and UTI history did not significantly differentiate resolved from unresolved cases.

Long-term monitoring is essential to prevent potential functional deterioration, particularly in high-risk patients. Due to the variability in clinical progression, clinicians should implement standardized follow-up protocols incorporating serial imaging and renal function assessments to ensure timely intervention.

## Figures and Tables

**Table 1:** Statistical summary.

Statistic	Mean	Median	Standard Deviation	Min	Max	25th Percentile	75th Percentile
Age at Diagnosis (months)	24.21	1.0	32.06	0.0	83.0	0.0	45.0
Age at First Admission (months)	31.31	11.04	34.34	0.33	106.93	2.27	51.31
Left Pelvis Diameter (mm)	11.87	11.0	6.02	4.0	27.0	8.0	14.0
Right Pelvis Diameter (mm)	13.96	12.0	7.79	3.0	28.0	9.0	15.0
Left Parenchyma Thickness (mm)	9.93	11.0	2.49	5.0	14.0	9.0	12.0
Right Parenchyma Thickness (mm)	9.54	10.0	2.1	4.0	12.0	9.0	11.0
Left Ureter Diameter (mm)	10.93	11.0	3.3	7.0	17.0	8.0	13.0
Right Ureter Diameter (mm)	11.81	10.0	4.15	7.0	21.0	9.0	15.0
Seric Urea (mg/dL)	21.57	23.0	8.27	7.0	33.0	13.0	28.0
Seric Creatinine (mg/dL)	0.32	0.31	0.1	0.12	0.5	0.26	0.36
GFR—Left Kidney (mL/min/1.73 m^2^)	64.27	63.0	19.61	36.0	108.0	49.0	76.0
GFR—Right Kidney (mL/min/1.73 m^2^)	61.55	55.0	25.09	9.1	115.0	47.0	78.0
Time to Resolution (months)	45.75	32.0	33.44	12.0	107.0	17.5	76.0
Follow-Up (months)	51.81	60.18	30.54	3.12	111.4	24.18	84.42

## Data Availability

The original contributions presented in this study are included in the article/Supplementary Material. Further inquiries can be directed to the corresponding author.

## Supplementary Materials

The following supporting information can be downloaded at: https://www.mdpi.com/article/10.3390/jcm14072463/s1.

## Abbreviations

The following abbreviations are used in this manuscript:

POM	Primary obstructive megaureter
VUR	Vesicoureteral reflux
VUJ	Vesicoureteral junction
UTI	Urinary tract infection
CT	Computed tomography
VCUG	Voiding cystourethrography
MRI	Magnetic resonance imaging
SFU	Society for Fetal Urology
DRF	Differential renal function
DTPA	Diethylenetriamine pentaacetate
AP	Anteroposterior
US	Ultrasound
HN	Hydronephrosis
IQR	Interquartile range
SD	Standard deviation
EAU	European Association of Urology
MRU	Magnetic resonance urography
