# Supplementary figures and images for: Predictive Factors for Spontaneous Resolution in Primary Obstructive Megaureter: The Impact of Hydronephrosis Severity on Clinical Outcomes
Source: J Clin Med. 2025 Apr 4;14(7):2463. doi: 10.3390/jcm14072463 (PMC11989593; doi:10.3390/jcm14072463)

## Slide 1
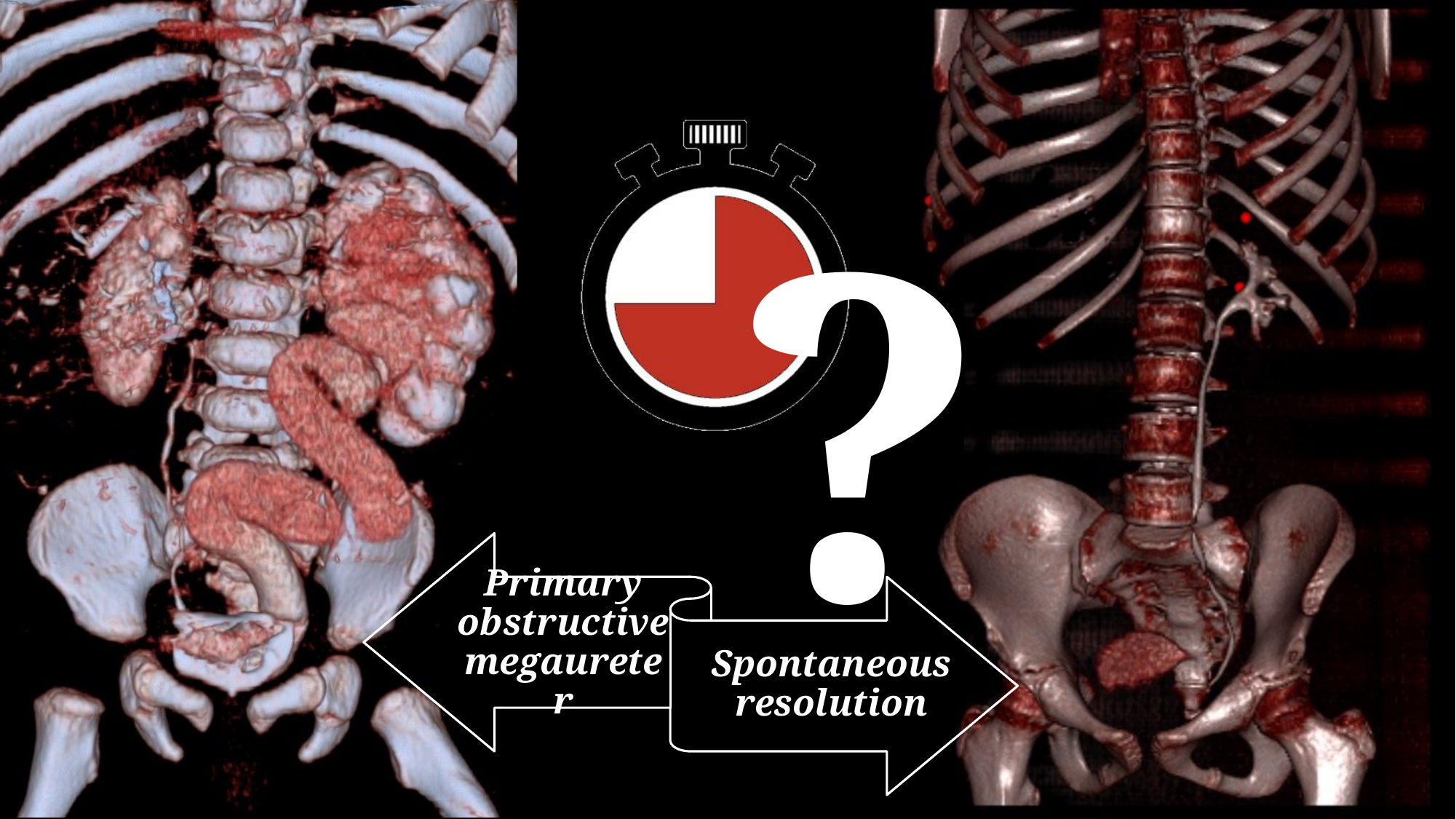

?

Supplement: Supplementary file 1 [file jcm-14-02463-s001.zip › jcm-3549959-supplementary.pptx]
